# Inflammatory risk of albumin combined with C-reactive protein predicts long-term cardiovascular risk in patients with diabetes

**DOI:** 10.18632/aging.205709

**Published:** 2024-03-29

**Authors:** Xiaoqun Xu, Houyong Zhu, Hanxin Wang, Xinyu Zhu, Libin Liu, Fengwei Zhang, Hongjuan Zhou, Mingzhi Zhu, Lingshan Dai, Long Cai

**Affiliations:** 1 Centre of Laboratory Medicine, Hangzhou Red Cross Hospital, Zhejiang, China; 2 Department of Cardiology, Hangzhou TCM Hospital Affiliated to Zhejiang Chinese Medical University, Hangzhou, Zhejiang, China; 3 The Fourth School of Clinical Medicine, Zhejiang Chinese Medical University, Hangzhou, Zhejiang, China; 4 Zhejiang University School of Medicine, Hangzhou, Zhejiang, China

**Keywords:** albumin, C-reactive protein, glasgow prognostic score, diabetes, cardiovascular death

## Abstract

Aims: The purpose of this study was to evaluate the predictive value of inflammatory risk as defined by the Glasgow Prognostic Score (GPS) for cardiovascular death in patients with diabetes.

Methods: This study included 4956 patients (≥18 years old) with diabetes in the National Health and Nutrition Survey from 1999 to 2010. The mortality rate was determined by the correlation with the national death index on December 31, 2019. The GPS was composed of the serum C-reactive protein and the albumin. The primary outcome was cardiovascular death and the secondary outcome was all-cause death. The Cox proportional risk model adjusted for demographic factors and traditional cardiovascular risk factors was used to analyze the cumulative risk of outcomes.

Results: Among 4956 diabetes patients with a median follow-up of 10.9 years, 601 cardiovascular deaths and 2187 all-cause deaths were recorded. After adequate model adjustment, compared with the low GPS group, the high GPS group (HR, 1.257 (1.007–1.570), *P* = 0.043) had a higher cardiovascular mortality. Compared with the low GPS group, the all-cause mortality of the high GPS group (HR, 1.394 (1.245–1.560), *P* < 0.001) was higher. The results of subgroup analyses were similar with that of the overall cohort.

Conclusions: The inflammatory risk as defined by the GPS was closely related to the increased risk of cardiovascular and all-cause death in patients with diabetes. It may be a convenient and efficient clinical practical risk assessment tool for patients with diabetes.

## INTRODUCTION

Diabetes is a major risk factor for cardiovascular disease (CVD), and CVD is also the most common cause of death in diabetes [1]. Compared with people without diabetes, their average life expectancy has decreased by about 10 years [2, 3]. It is estimated that about 7.8% of Americans currently have diabetes, but 38.0% of adults are in pre-diabetes state [4] The prevalence of diabetes in China is also increasing rapidly. From 2013 to 2018, the prevalence of diabetes in China increased from 10.9% to 12.4% [5] In addition to routine lifestyle and drug management, how to effectively identify changeable risk factors is very important to prevent or delay complications and premature death of diabetes [6, 7] The COLCOT [8] and CANTOS [9] trials confirmed that in patients with coronary artery disease accompanied by low-grade inflammation, in addition to conventional secondary prevention, anti-inflammatory treatment could further reduce the risk of cardiovascular events, which means the importance of inflammation in atherosclerosis. Previous studies [10–12] found that the inflammatory risk of Glasgow Prognostic Score (GPS) defined albumin combined with C-reactive protein (CRP) predicted poor prognosis in patients with acute myocardial infarction (AMI), and its predictive ability was comparable to Global Registry of Acute Coronary Events (GRACE) score. Patients with diabetes are also in a long-term low-grade inflammatory state, but whether this score could equally effectively predict the long-term prognosis of them has not been reported yet. Therefore, in this study, we analyzed the data of six periods of the National Health and Nutrition Survey (NHANES) from 1999 to 2010, and assessed the predictive value of inflammatory risk defined by the GPS on cardiovascular death in patients with diabetes through the correlation with the national death index (NDI) on December 31, 2019.

## MATERIALS AND METHODS

### National health and nutrition examination survey

The NHANES is a large, multistage, nationally representative survey of the US civilian non-institutionalized population conducted by the National Center for Health Statistics (NCHS). Since 1999, it has become a continuous project, representing a cycle every two years. Each survey participant shall complete a family interview and receive a physical examination in a mobile physical examination center. A detailed description of the NHANES method is published elsewhere [13, 14] NHANES is approved by the Institutional Review Committee and includes written informed consent. More detailed information can be found at http://www.cdc.gov/nchs/nhanes/irba98.htm.

**Figure 1:**
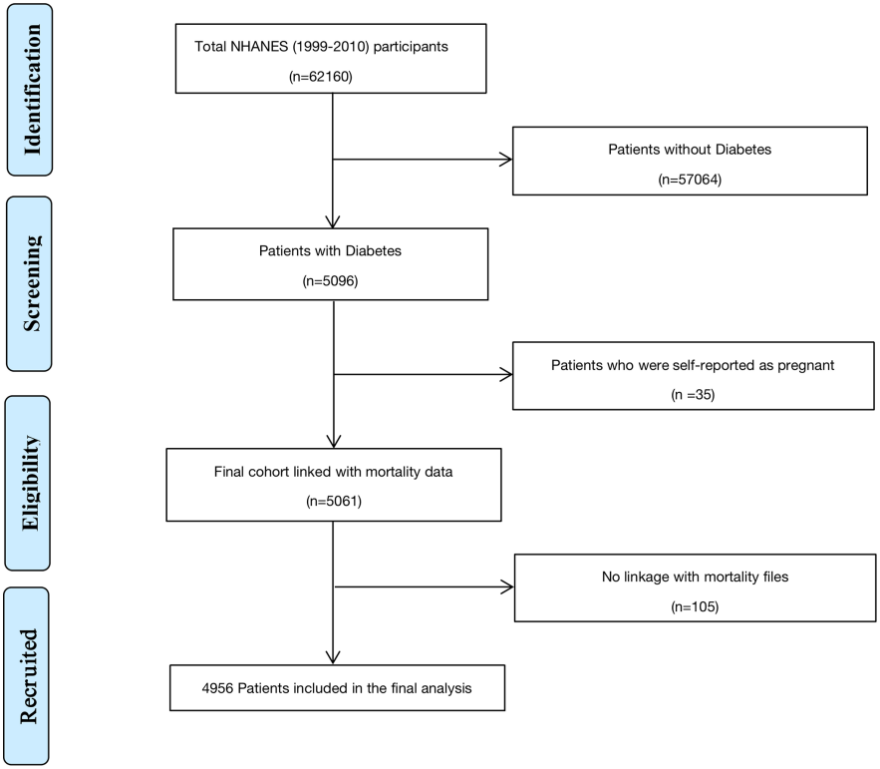
Flow diagram for recruitment of patients.

This study collected data from six cycles of NHANES (1999–2010), while CRP data was missing or the detection method was inconsistent in four cycles of 2011–2018, so it was excluded. The initial indexing time was March 1999. Diabetes was determined to be diagnosed by a self-reported doctor, using insulin or oral hypoglycemic drugs, with fasting blood glucose ≥7.0 mmol/L, or glycosylated hemoglobin ≥6.5%. After excluding the non compliant subjects, 5096 diabetes participants were left as the subjects. After excluding 35 self-reported pregnancies at baseline, 5061 participants were initially included in the current study. Through the correlation with the national death index on December 31, 2019, the death status of these participants was determined, including cardiovascular death, all-cause death, cerebrovascular death, and cancer death [15] After excluding 105 individuals whose death status could not be determined, the remaining 4956 diabetes patients were finally enrolled in this study (Figure 1).

### Baseline data

The standardized questionnaire was used to collect information about age, gender, race, education level, smoking, drinking, and poverty income ratio (PIR) from family interviews. Weight and height were obtained from physical examination, and body mass index (BMI) was calculated as weight divided by the square of height. Race was divided into non-Hispanic white and non-white. The education level was divided into < high school and ≥ high school. Poverty income ratio (PIR) was divided into <1.5, 1.5 - 3.37, and >3.37. Blood glucose, glycosylated hemoglobin, triglyceride and total cholesterol, as well as other related biochemical indicators were obtained from the biochemical indicators examined by NHANES laboratory. Through the questionnaire, we also obtained the history of asthma, emphysema, chronic bronchitis, arthritis, liver disease, cancer and other diseases. The specific diagnosis basis could be found in the Supplementary Appendix 1.

### Measurement of serum C-reactive protein and albumin

The agreed participants collected blood through venipuncture. The vials were stored under appropriate freezing conditions (–20°C) and then sent to the University of Washington for testing. CRP was quantified by latex-enhanced turbidimetry. Albumin was detected by the bromocresol purple method. Specific measurement methods can be found in the NHANES Laboratory Procedure Manual [14].

### Grading of inflammatory risk defined by Glasgow prognostic score

Participants with a low CRP level (≤10 mg/L) and a high albumin level (≥35 g/L) were designated as 0 points for GPS. One or two anomalies of these two parameters are designated as 1 point for GPS.

### Outcome events

We used death certificate information available from the NDI through December 31, 2019. Matches to NHANES and NDI were made by identifying unique individual sequence numbers (SEQNs). The primary outcome was cardiovascular death. The secondary outcome was all-cause death, which was defined as death from any cause. Other complementary outcomes included cerebrovascular death and cancer death. The main causes of death were classified according to the International Statistical Classification of Diseases and Related Health Problems, 10^th^ Revision (ICD 10), and the standardized list of codes (UCOD_LEADING) created by NCHS. The cardiovascular death code is 001, the cerebrovascular death code is 005, and the cancer death code is 002. More information is available at https://www.cdc.gov/nchs/data/datalinkage/public-use-linked-mortality-files-data-dictionary.pdf

### Statistical analysis

We compared the baseline characteristics grouped by inflammatory risk defined by the GPS by the following methods: Wilcoxon test was used for the median and quartile of continuity variables, and the Pearson chi-square was used for the test of categorical variables. The missing values of continuous variables were filled by the expectation maximization (EM) method, while the missing values of classified variables were filled by adding a group of missing values. Based on our assessment of the possibility of covariates as confounding factors in the relationship between the GPS and outcomes events, the Cox proportional risk model in the stepwise inclusion model was used to estimate the survival analysis. The relevant confounding variables for adjustment include demographic factors (age, gender, race, education level, smoking, drinking, PIR, and BMI) and traditional cardiovascular risk factors (glycosylated hemoglobin, hyperlipidemia, hypertension, CVD, chronic pulmonary disease, arthritis, liver disease, cancer, and moderate to severe kidney disease). Model 1 was adjusted by basic demographic factors (age, gender, and race); Model 2 was adjusted by adding education level, smoking, PIR, and BMI to model 1; Model 3 was adjusted by adding the above traditional cardiovascular risk factors to Model 2 as our fully adjusted model. The hazard ratio (HR) and corresponding 95% confidence interval (CI) were obtained from Cox proportional risk model 3, and the cumulative risk standard plot of outcomes was established based on this model. Through visual evaluation of the cumulative risk standard plot and the logarithm of negative logarithm of Cox survival function, it was confirmed that the main research variable did not have time-dependent effects, thus verifying the hypothesis of Cox model.

We performed subgroup analyses on the basis of fully adjusted model (Model 3) according to age, gender, race, smoking, drinking, hypertension, hyperlipidemia, CVD, chronic pulmonary disease, arthritis, cancer, liver disease, moderate and severe kidney disease, and follow-up time.

Other inflammatory scores of concern, such as platelet to lymphocyte ratio (PLR) and neutrophil to lymphocyte ratio (NLR), were also considered to have good predictive value for the risk of cardiovascular adverse events [16–19]. Therefore, we conducted a post-hoc analysis to evaluate the long-term prognosis of the PLR and NLR for cardiovascular outcomes and other secondary outcomes. Both the PLR and NLR scores were binary variables with a median cutoff point. Below the median, they were defined as the low-risk (0) group, and above the median, they were defined as the high-risk (1) group. HR (95% CI), number, and median (quartile) were taken as summary statistics in corresponding cases. Bilateral *P*-values < 0.05 were considered statistically significant. The data were analyzed using SPSS 26.0 (SPSS, Inc., Chicago, IL, USA).

### Availability of data and material

Publicly available datasets were analyzed in this study. This data can be found here: https://www.cdc.gov/nchs/nhanes/index.htm.

## RESULTS

### Patient characteristics

A total of 4956 diabetes subjects were finally included in this study (Figure 1). The median age was 64 years old, 49.6% were female, 25.2% were non-Hispanic white people, and the median follow-up time was 10.9 years (Table 1). There were 3431 (69.2%) patients with hypertension, 3241 (65.4%) with hyperlipidemia, 1367 (27.6%) with CVD, 994 (20.1%) with chronic pulmonary disease, 2210 (44.6%) with arthritis, 669 (13.5%) with cancer, 283 (5.7%) with hepatic insufficiency, and 1148 (23.2%) with moderate and severe kidney disease. The missing values were filled through the EM method, which can be seen in Supplementary Table 1.

**Table 1:** Baseline characteristics according to GPS in NHANES 1999-2010.

**Characteristics**	**GPS**		***P*-value**
	**0 (*N* = 4082)**	**1 (*N* = 874)**	
CRP, median (quartile), mg/L	2.9 (1.3–5.5)	15.1 (11.6–21.6)	<0.001
ALB, median (quartile), g/L	42.0 (40.0–44.0)	39.0 (36.0–41.0)	<0.001
Age, median (quartile), years	64 (53.0–73.0)	60 (49.0–69.3)	<0.001
Female, no. (%)	1924 (47.1)	535 (61.2)	<0.001
Race, no. (%)			<0.001
Non-Hispanic white	977 (23.9)	274 (31.4)	
Non-white	3105 (76.1)	600 (68.6)	
Education status, no. (%)			0.480
≥High school	2288 (56.0)	485 (55.5)	
<High school	1754 (43.0)	381 (43.6)	
Smoking, no. (%)			0.018
Never	1951 (47.8)	404 (46.2)	
Current/former	2097 (51.4)	462 (52.8)	
Alcohol consumption, no. (%)			0.027
Consumed alcohol	2161 (52.9)	455 (52.1)	
Did not consume alcohol	1383 (33.9)	328 (37.5)	
BMI, median (quartile), kg/m^2^	30.1 (26.7–34.2)	34.9 (29.8–40.7)	<0.001
PIR, no. (%)			<0.001
≤1	1486 (36.4)	381 (43.6)	
1–3	1231 (30.2)	251 (28.7)	
>3	950 (23.3)	159 (18.2)	
HbA1c, no. (%)			<0.001
<7.0	2250 (55.1)	419 (47.9)	
≥7.0	1832 (44.9)	455 (52.1)	
Hypertension, no. (%)	2805 (68.7)	626 (71.6)	0.174
Hyperlipidemia, no. (%)	2700 (66.1)	541 (61.9)	0.017
CVD, no. (%)	1112 (27.2)	255 (29.2)	0.465
CPD, no. (%)	754 (18.5)	240 (27.5)	<0.001
Arthritis, no. (%)	1762 (43.2)	448 (51.3)	0.000
Cancer, no. (%)	569 (13.9)	100 (11.4)	0.123
Liver dysfunction, no. (%)	216 (5.3)	67 (7.7)	0.022
Moderate or severe nephropathy, no. (%)	915 (22.4)	233 (26.7)	0.007
Medication use			<0.001
No insulin or pills	532 (13.0)	99 (11.3)	
Only diabetes pills	1857 (45.5)	336 (38.4)	
Only insulin	396 (9.7)	108 (12.4)	
Pills and insulin	347 (8.5)	94 (10.8)	
Follow-up length, years			0.638
<10	1595 (39.1)	349 (39.9)	
≥10	2487 (60.9)	525 (60.1)	

Baseline characteristics according to GPS in NHANES 1999–2010. Abbreviations: ALB: albumin; BMI: body mass index; CPD: chronic pulmonary disease; CVD: cardiovascular disease; GPS: Glasgow Prognostic Score; HbA1c: glycated hemoglobin; PIR: poverty income ratio. Values are numbers (%) or medians (quartile).

### Primary outcome

**Figure 2:**
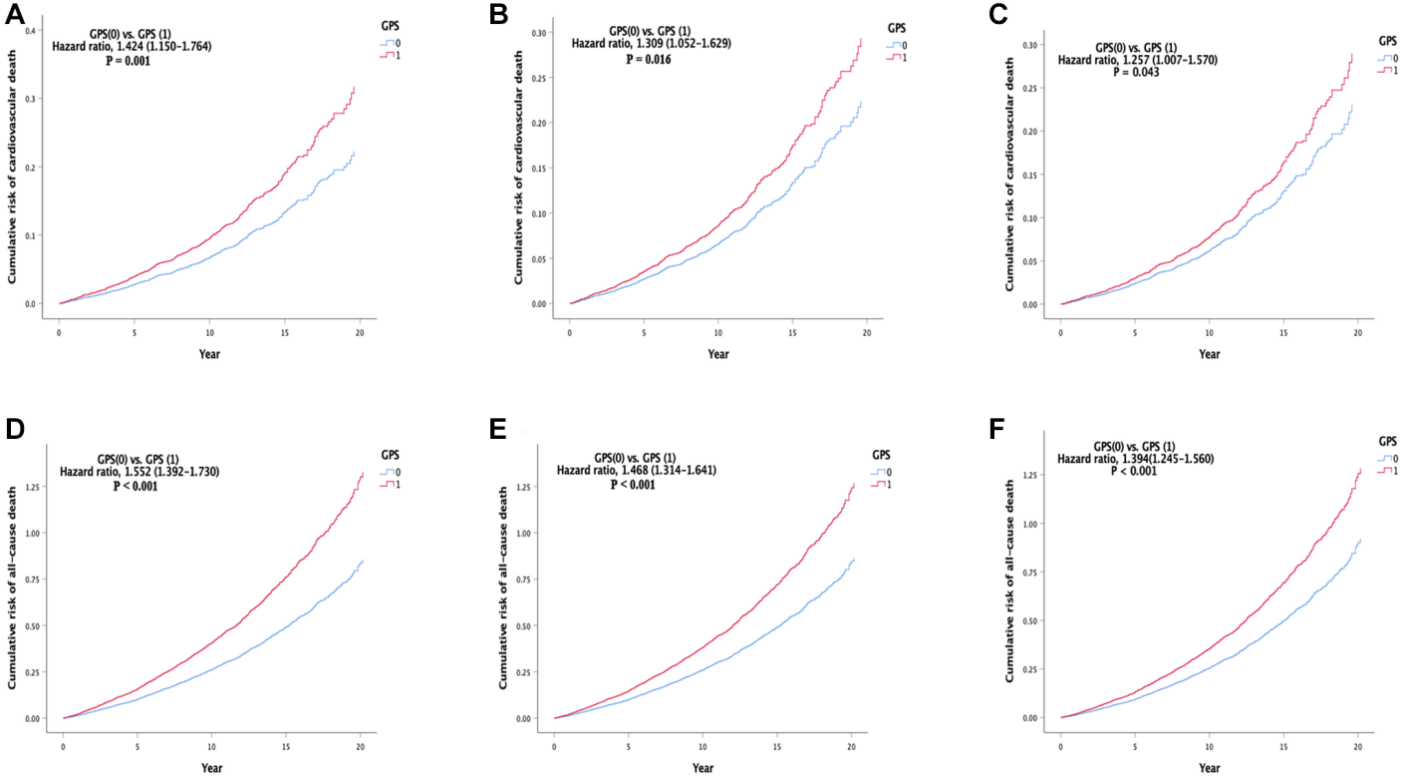
**Cumulative incidence of the primary and second outcomes in different models.** (**A**) Cumulative risk of cardiovascular death in model 1. (**B**) Cumulative risk of cardiovascular death in model 2. (**C**) Cumulative risk of cardiovascular death in model 3. (**D**) cumulative risk of all-cause death in model 1. (**E**) Cumulative risk of all-cause death in model 2. (**F**) Cumulative risk of all-cause death in model 3. Abbreviation: GPS: Glasgow Prognostic Score.

In Model 1, compared with the low GPS group, the high GPS group (HR, 1.424 (1.150–1.764), *P* = 0.001) had a higher cardiovascular mortality (Table 2 and Figure 2A). In Model 2, compared with the low GPS group, the high GPS group (HR, 1.309 (1.052–1.629), *P* = 0.016) had a higher cardiovascular mortality (Table 2 and Figure 2B). In the fully adjusted model (Model 3), compared with the low GPS group, the high GPS group (HR, 1.257 (1.007–1.570), *P* = 0.043) had a higher cardiovascular mortality (Table 2 and Figure 2C). The results of the three models showed consistency.

**Table 2:** Cox regression analysis for the primary and secondary outcomes.

**Outcomes**	**GPS, HR (95% Cl)**	
	**0**	**1**
Cardiovascular mortality		
Model 1	1.000 (Reference)	1.424 (1.150–1.764)
* P*-Value		0.001
Model 2	1.000 (Reference)	1.309 (1.052–1.629)
* P*-Value		0.016
Model 3	1.000 (Reference)	1.257 (1.007–1.570)
* P*-Value		0.043
All-cause mortality		
Model 1	1.000 (Reference)	1.552 (1.392–1.730)
* P*-Value		<0.001
Model 2	1.000 (Reference)	1.468 (1.314–1.641)
* P*-Value		<0.001
Model 3	1.000 (Reference)	1.394 (1.245–1.560)
* P*-Value		<0.001

Cox regression analysis for the primary and secondary outcomes. Model 1 is adjusted for age, gender, and race. Model 2 is adjusted for variables in Model 1 + education status, smoking, drinking, poverty-income ratio, and body mass index. Model 3 is adjusted for variables in Model 2 + glycated hemoglobin, cardiovascular disease, hyperlipidemia, hypertension, chronic pulmonary disease, liver disease, arthritis, cancer, and moderate to severe nephropathy. Abbreviations: CI: confidence interval; GPS: Glasgow Prognostic Score; HR: hazard ratio.

### Secondary outcome

In Model 1, compared with the low GPS group, the all-cause mortality of the high GPS group (HR, 1.552 (1.392–1.730), *P* < 0.001) was higher (Table 2 and Figure 2D). In Model 2, compared with the low GPS group, the all-cause mortality of the high GPS group (HR, 1.468 (1.314–1.641), *P* < 0.001) was higher (Table 2 and Figure 2E). In the fully adjusted model (Model 3), compared with the low GPS group, the all-cause mortality of the high GPS group (HR, 1.394 (1.245–1.560), *P* < 0.001) was higher (Table 2 and Figure 2F). In addition, there was no significant statistical difference between the GPS groups in the cerebrovascular death, while the cancer death was higher in the high GPS group (Supplementary Table 2 and Supplementary Figure 1). All secondary outcomes were consistent in all models.

### Subgroup analyses

In the subgroups determined according to age, gender, race, smoking, drinking, hypertension, hyperlipidemia, CVD, chronic pulmonary disease, arthritis, cancer, liver disease, moderate and severe kidney disease, and 10-year follow-up period, the impact of GPS on the primary and secondary outcomes was almost identical (Table 3). Further interaction tests showed that the risk of cardiovascular death assessed by the GPS was different in chronic pulmonary disease and follow-up time.

**Table 3:** Subgroup analyses of the effect of GPS on the primary and secondary outcomes.

**Subgroup**	**Cardiovascular mortality**		***P* for interaction**	**All-cause mortality**		***P* for interaction**
	**GPS, HR (95% Cl)**			**GPS, HR (95% Cl)**		
Age	0	1		0	1	
≥65 years	1.000 (Reference)	1.383 (1.060−1.805)	0.417	1.000 (Reference)	1.390 (1.207−1.601)	0.440
<65 years	1.000 (Reference)	0.856 (0.568−1.288)		1.000 (Reference)	1.202 (0.990−1.458)	
Gender
Male	1.000 (Reference)	1.102 (0.795−1.526)	0.476	1.000 (Reference)	1.379(1.174−1.619)	0.885
Female	1.000 (Reference)	1.508 (1.105−2.057)		1.000 (Reference)	1.453(1.239−1.705)	
Race
Non-Hispanic white	1.000 (Reference)	1.193 (0.846−1.647)	0.548	1.000 (Reference)	1.471(1.246−1.737)	0.800
Non-white	1.000 (Reference)	1.328 (0.975−1.809)		1.000 (Reference)	1.139 (1.003−1.294)	
Education
≥High school	1.000 (Reference)	1.191 (0.860−1.649)	0.283	1.000 (Reference)	1.358 (1.152−1.601)	0.723
<High school	1.000 (Reference)	1.301 (0.955−1.771)		1.000 (Reference)	1.426 (1.218−1.670)	
Smoking
Never	1.000 (Reference)	1.315 (1.002−1.727)	0.567	1.000 (Reference)	1.297 (1.083−1.554)	0.673
Current/former smokers	1.000 (Reference)	1.303 (0.971−1.748)		1.000 (Reference)	1.474 (1.274−1.706)	
Alcohol consumption
Consumed alcohol	1.000 (Reference)	1.219 (0.891−1.668)	0.152	1.000 (Reference)	1.414 (1.207−1.657)	0.116
Did not consume alcohol	1.000 (Reference)	1.561 (1.097−2.222)		1.000 (Reference)	1.528 (1.267−1.843)	
HbA1C
<7.0	1.000 (Reference)	1.249 (0.888−1.758)	0.774	1.000 (Reference)	1.654 (1.405−1.946)	0.054
≥7.0	1.000 (Reference)	1.296 (0.961−1.747)		1.000 (Reference)	1.222(1.043−1.430)	
Hypertension
Yes	1.000 (Reference)	1.170 (0.908−1.508)	0.343	1.000 (Reference)	1.320 (1.159−1.503)	0.125
No	1.000 (Reference)	1.769 (1.103−2.836)		1.000 (Reference)	1.739 (1.375−2.200)	
Hyperlipidemia
Yes	1.000 (Reference)	1.172 (0.883−1.557)	0.544	1.000 (Reference)	1.282 (1.108−1.482)	0.130
No	1.000 (Reference)	1.444 (1.003−2.081)		1.000 (Reference)	1.653 (1.376−1.985)	
CVD
Yes	1.000 (Reference)	1.368 (0.999−1.874)	0.364	1.000 (Reference)	1.561 (1.310−1.860)	0.107
No	1.000 (Reference)	1.090 (0.794−1.496)		1.000 (Reference)	1.287 (1.109−1.494)	
Chronic pulmonary disease
Yes	1.000 (Reference)	0.678 (0.420−1.094)	0.003	1.000 (Reference)	1.091 (0.875−1.360)	0.028
No	1.000 (Reference)	1.578 (1.229−2.027)		1.000 (Reference)	1.539 (1.350−1.756)	
Arthritis
Yes	1.000 (Reference)	1.191 (0.883−1.606)	0.469	1.000 (Reference)	1.072 (0.945−1.217)	0.378
No	1.000 (Reference)	1.334 (1.029−1.730)		1.000 (Reference)	1.159 (1.016−1.323)	
Cancer
Yes	1.000 (Reference)	1.676 (0.939−2.992)	0.827	1.000 (Reference)	1.414 (1.066−1.876)	0.967
No	1.000 (Reference)	1.205 (0.944−1.536)		1.000 (Reference)	1.389 (1.227−1.573)	
Liver dysfunction
Yes	1.000 (Reference)	1.035 (0.363−2.957)	0.149	1.000 (Reference)	1.540 (1.012−2.346)	0.088
No	1.000 (Reference)	1.229 (0.976−1.549)		1.000 (Reference)	1.348 (1.197−1.517)	
Moderate or severe nephropathy
Yes	1.000 (Reference)	1.422 (1.019−1.984)	0.198	1.000 (Reference)	1.443 (1.208−1.722)	0.503
No	1.000 (Reference)	1.134 (0.838−1.535)		1.000 (Reference)	1.356 (1.170−1.572)	
Follow-up periods
<10 years	1.000 (Reference)	1.300 (0.998−1.694)	<0.001	1.000 (Reference)	1.564 (1.370−1.787)	<0.001
≥10 years	1.000 (Reference)	1.138 (0.754−1.717)		1.000 (Reference)	1.186 (0.954−1.474)	

Subgroups analyses of the effect of GPS on the primary and secondary outcomes. Abbreviations: CI: confidence interval; GPS: Glasgow Prognostic Score; HR: hazard ratio.

### Post-hoc analysis

In the fully adjusted model (Model 3), compared to the low PLR group, there was no statistically significant difference in cardiovascular mortality (HR, 0.969 (0.823–1.140), *P* = 0.704) in the high PLR group, but all-cause mortality (HR, 0.913 (0.839–0.995), *P* = 0.037) was lower. There were no statistically significant differences between the two groups in cerebrovascular death (HR, 0.718 (0.514–1.004), *P* = 0.053) and cancer death (HR, 1.003 (0.813–1.239), *P* = 0.975) (Supplementary Table 3). Compared with the low NLR group, the high NLR group had higher cardiovascular mortality (HR, 1.455 (1.229–1.722), *P* < 0.001), all-cause mortality (HR, 1.516 (1.388–1.657), *P* < 0.001), and cancer mortality (HR, 1.314 (1.058–1.632), *P* = 0.014), but there was no significant statistical difference in cerebrovascular mortality (HR, 0.993 (0.707–1.395), *P* = 0.968) between the two groups (Supplementary Table 3).

## DISCUSSION

In this nationwide representative cohort study of diabetes population, after fully adjusting for confounding factors, the high level of inflammatory risk defined by the GPS had an increased risk of cardiovascular death and all-cause death. The results of subgroup analyses were similar to that of the overall cohort. This may be a very important discovery. First, it showed that only the initial inflammatory risk defined by the GPS effectively predicted the long-term prognosis in patients with diabetes. Second, this also provided some evidence for the anti-inflammatory treatment of diabetes.

According to the existing evidence and scientific statement of the American Diabetes Association (ADA), the prevention strategies for diabetes patients to reduce the risk of CVD include physical activity, nutrition, weight, smoking cessation, blood sugar, blood pressure, blood lipids and other lifestyle and drug management [20–23]. However, inflammation, like lipid, is essential to the occurrence and development of CVD. Chronic subclinical inflammation is the key process of CVD. Although the role of lipid-lowering drugs in the prevention and treatment of CVD has been established by extensive research in the past decades, the regulation of inflammation is a positive controversial topic. In recent years, many studies [24–26] found that inflammation played an important role in the residual cardiovascular risk. Liu et al. [24] found that the increase of CRP in patients with chronic coronary syndrome treated with statins was related to the increased risk of major cardiovascular adverse events (MACEs). Oikonomou et al. [25] predicted the residual cardiovascular risk by non-invasive detection of coronary artery by computed tomography, and found that the inflammatory state of coronary artery was significantly related to the increased risk of cardiovascular death.

Patients with diabetes are at risk of low-grade inflammation for a long time. Both insulin resistance in type 2 diabetes and immune mediated destruction of pancreatic β cells in type 1 diabetes produce proinflammatory cytokines, such as tumor necrosis factor α and interleukin-6 [27, 28]. Low-grade inflammation is also considered as a risk factor for cardiovascular events and all-cause death in patients with diabetes [27, 29]. In addition to CRP reflecting inflammatory status, albumin has also been found to have multiple binding sites that provide an ideal platform for scavenging free radicals, endowing it with powerful anti-inflammatory and antioxidant properties, and it also combines various inflammatory mediators to participate in regulating the immune response in systemic inflammation, and it is associated with the pathogenesis and complications of diabetes [30–32].

The GPS was first proposed by Forrest et al., who found that the GPS was effective in predicting the survival of non-small cell lung cancer patients [33]. In recent years, some studies found that it also had predictive value for the survival of AMI [10–12, 34]. Our previous study [11] compared different versions of GPS using receiver operating curve (ROC), and this present version of GPS could more effectively predict cardiovascular risk in patients with AMI, which was used as the scoring standard for this study. The present study found that patients with a high risk of inflammation had an increased risk of cardiovascular death, suggesting that the initial assessment of the GPS had an important impact on the long-term prognosis of diabetes patients. In the recently released randomized controlled trials (RCTs) of anti-inflammatory treatment, the COLCOT and CANTOS trials [8, 9] showed that the patients with myocardial infarction with high baseline CRP levels reduced the risk of MACEs through anti-inflammatory treatment, but the baseline CRP of patients with myocardial infarction included in the CIRT trial [35] was only 1.6 mg/L, and the results showed that anti-inflammatory treatment did not improve the prognosis of patients. Therefore, reducing the residual cardiovascular risk by adding anti-inflammatory drugs to conventional cardiovascular treatment is expected to become a transformation mode of CVD and diabetes management. In addition, the risk of all-cause death also increased with a high inflammatory risk, which is partly due to the increased risk of cardiovascular and cancer death. The correlation between cancer death and inflammatory risk may be related to the inflammatory response of cancer cells to the destruction of local peripheral tissues and long-term energy consumption [36, 37]. Since this article mainly discusses the impact of inflammatory risk on cardiovascular risk in diabetes population, cancer death will not be mainly discussed.

In subgroup analyses, the trend of all results was similar with the overall cohort. The inter-group comparison found that the inflammatory risk could more effectively predict the risk of cardiovascular death in patients without chronic pulmonary disease than in patients with chronic pulmonary disease, which may be in a long-term inflammatory state with asthma and chronic bronchitis, which may interfere with the predicted value of this inflammatory risk. In addition, the GPS defined inflammatory risk had a higher predictive ability for patients with a median follow-up of less than 10 years. After all, it is not easy for laboratory indicators tested once to have a role in long-term prognosis. The post-hoc analysis showed that the GPS and NLR had better predictive value for long-term cardiovascular death risk of diabetes compared with the PLR. In the definition of the NLR score, the selection of its cutoff value was not entirely the same in previous studies [16–19], and further research was needed to clarify its cutoff value for clinical use. The GPS had a very clear and convenient definition, which may have higher clinical practical value.

There are some limitations for this study. The diabetes cohort was collected by NHANES staff or partners, and there was no diabetes classification in the database. Secondly, some basic diseases of interest, such as systemic lupus erythematosus, should ideally be included in the model, but also not in the database. In addition, the aim of this study was to explore the impact of baseline inflammatory risk as defined by the GPS on cardiovascular risk in patients with diabetes, but there was a lack of dynamic monitoring of its level.

## CONCLUSIONS

The inflammatory risk as defined by the GPS is closely related to the risk of cardiovascular and all-cause death in diabetes patients, its high-level leads to an increase in the risk of cardiovascular and all-cause death. It may be a convenient and efficient clinical practical risk assessment tool for patients with diabetes. However, large-scale and prospective clinical trials still need to be carried out to evaluate the effectiveness of this inflammatory risk and further test whether reducing its level reduce cardiovascular risk.

## Supplementary Materials

Supplementary Appendix 1

Supplementary Figure 1

Supplementary Tables
