# Supplementary material for: Inflammatory risk of albumin combined with C-reactive protein predicts long-term cardiovascular risk in patients with diabetes
Source: Aging (Albany NY). 2024 Mar 29;16(7):6348–63. doi: 10.18632/aging.205709 (PMC11042957; doi:10.18632/aging.205709)
Supplement: Supplementary Appendix 1 [file aging-16-205709-s001.pdf]

SUPPLEMENTARY APPENDIX 1

Diagnostic basis for diseases included in this study

Hypertension

Includes history of hypertension diagnosed by the doctor, high systolic blood pressure (≥140 mmHg) or high diastolic blood pressure (≥90 mmHg), use of antihypertensive drugs, or final death due to hypertension.

Hyperlipidemia

Includes total cholesterol ≥5.7 mmol/L, or triglyceride ≥1.65 mmol/L.

Cardiovascular disease

Cardiovascular disease is identified as a combination of standardized medical status questionnaires administered during self-reported physician diagnosis and personal interviews, including congestive heart failure, coronary heart disease, angina pectoris, heart attack, and stroke.

Chronic pulmonary disease

Chronic pulmonary disease is identified as a combination of standardized medical status questionnaires administered during self-reported physician diagnosis and personal interviews, including asthma, emphysema, and chronic bronchitis.

Arthritis

Arthritis is identified as a combination of standardized medical status questionnaires administered during self-reported physician diagnosis and personal interviews.

Cancer

Cancer is identified as a combination of standardized medical status questionnaires administered during self-reported physician diagnosis and personal interviews.

Liver dysfunction

Liver dysfunction is identified as a combination of standardized medical status questionnaires administered during self-reported physician diagnosis and personal interviews, and the value of alanine aminotransferase ≥150 U/L.

Moderate or severe nephropathy

Moderate/severe nephropathy is defined as GFR <60 min × 1.73 m^2, the calculation of GFR refers to the method of Levey et al. (https://doi.org/10.7326/0003-4819-130-6-199903160-00002).
