# Supplementary material for: Inflammatory risk of albumin combined with C-reactive protein predicts long-term cardiovascular risk in patients with diabetes
Source: Aging (Albany NY). 2024 Mar 29;16(7):6348–63. doi: 10.18632/aging.205709 (PMC11042957; doi:10.18632/aging.205709)
Supplement: Supplementary Figure 1 [file aging-16-205709-s002.pdf]

SUPPLEMENTARY FIGURE

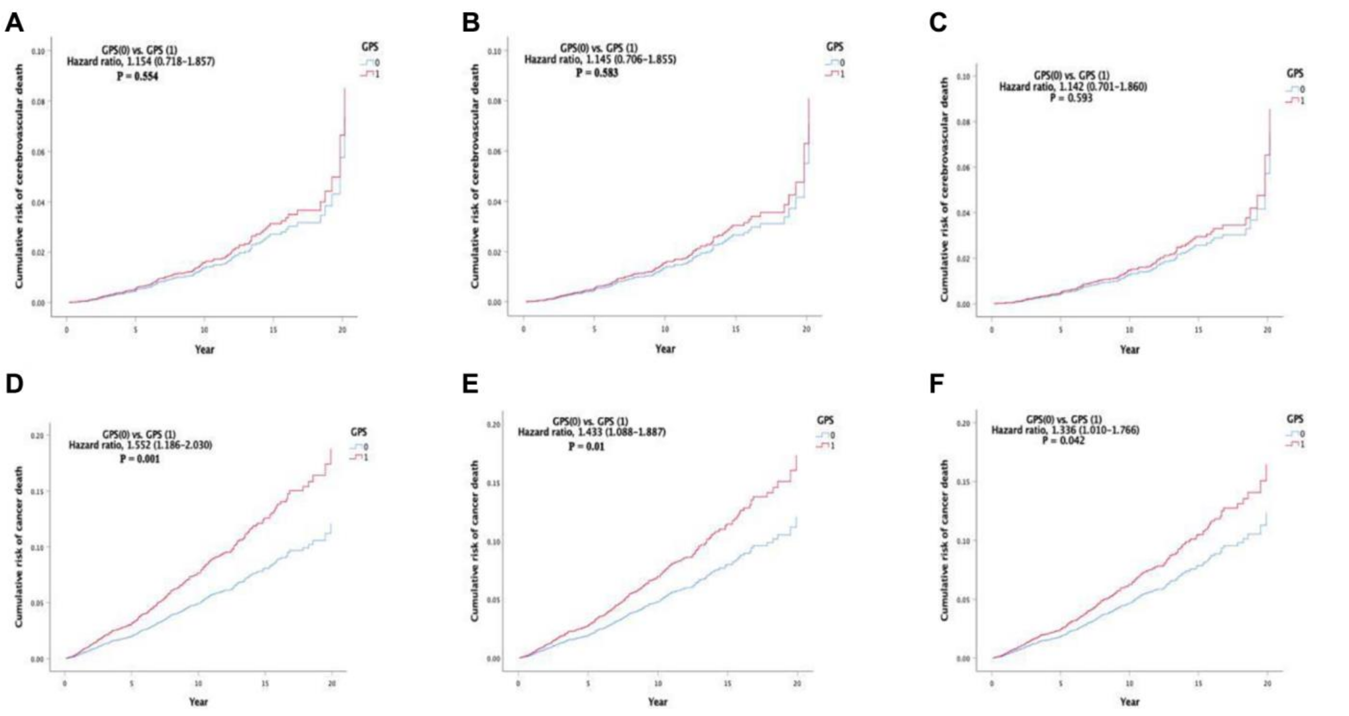

Supplementary Figure 1. Cumulative incidence of the supplemental secondary outcomes in different models 3. (**A**) Cumulative risk of cerebrovascular death in model 1. (**B**) Cumulative risk of cerebrovascular death in model 2. (**C**) Cumulative risk of cerebrovascular death in model 3. (**D**) cumulative risk of cancer death in model 1. (**E**) Cumulative risk of cancer death in model 2. (**F**) Cumulative risk of cancer death in model 3. Abbreviation: GPS: Glasgow Prognostic Score.
