# Supplementary material for: Inflammatory risk of albumin combined with C-reactive protein predicts long-term cardiovascular risk in patients with diabetes
Source: Aging (Albany NY). 2024 Mar 29;16(7):6348–63. doi: 10.18632/aging.205709 (PMC11042957; doi:10.18632/aging.205709)
Supplement: Supplementary Tables [file aging-16-205709-s003.pdf]

SUPPLEMENTARY TABLES

Supplementary Table 1. Statistics of missing value and extremesa.

	N	Mean	Std. deviation	Missing		No. of extremes[a]	
				Count	Percent	Low	High
PERMTH_INT	4956	129.47	59.07	0	0	112	12
BMI, kg/m^2	4562	31.81	7.273	394	7.9	5	181
CRP, mg/L	4499	6.50	11.834	457	9.2	0	119
Age, years	4956	61.96	14.348	0	0	198	0
Glucose, mmol/l	4452	8.48	3.942	504	10.2	0	231
HbA1c, no.	4548	7.26	1.786	408	8.2	0	272
HDL, mmol/L	4471	1.24	0.370	485	9.8	12	192
Serum albumin, g/L	4452	41.29	3.471	504	10.2	140	60
ALT, U/L	4439	27.29	24.830	517	10.4	0	115
BUN, mmol/L	4450	5.84	3.090	506	10.2	0	182
Tch, mmol/L	4451	5.03	1.218	505	10.2	20	154
TG, mmol/L	4448	2.26	2.172	508	10.3	0	108
Creatinine, μ mol/L	4451	88.90	60.865	505	10.2	0	83
Cardiovascular death, no.	4956			0	0		
All-cause death, no.	4956			0	0		
Cerebrovascular death, no.	4956			0	0		
Cancer death, no.	4956			0	0		
Smoking, no.	4914			42	0.8		
Hypertension, no.	4944			12	0.2		
Gender, no.	4956			0	0		
Education status, no. (%)	4908			48	1		
PIR, no.	4956			0	0		
Race, no.	4956			0	0		
Insulin, no.	4921			35	0.7		
Diabetes pills, no.	3771			1185	23.9		
Asthma, no.	4952			4	0.1		
Arthritis, no.	4912			44	0.9		
CVD	4921			35	0.7		
Emphysema, no.	4909			47	0.9		
Chronic bronchitis, no.	4906			50	1.0		
Liver dysfunction, no.	4901			55	1.1		
Cancer, no.	4909			47	0.9		
Alcohol consumption, no.	4956			0	0		

[a]Number of cases outside the range (Q1 − 1.5 × IQR, Q3 + 1.5 × IQR). Abbreviations: ALT: alanine aminotransferase; BMI: body mass index; BUN: blood urea nitrogen; CRP: C reactive protein; CVD: cardiovascular disease; HbA1c: glycated hemoglobin; HDL: high-density lipoprotein; PIR: poverty-income ratio; Tch: total cholesterol; TG: triglyceride.

Supplementary Table 2. Cox regression analysis for the supplementary secondary outcomes.

Outcomes	GPS, HR (95% Cl)	
	0	1
Cerebrovascular mortality		
Model 1	1.000 (Reference)	1.154 (0.718–1.857)
P-Value		0.554
Model 2	1.000 (Reference)	1.145 (0.706–1.855)
P-Value		0.583
Model 3	1.000 (Reference)	1.142 (0.701–1.860)
P-Value		0.593
Cancer mortality		
Model 1	1.000 (Reference)	1.552 (1.186–2.030)
P-Value		0.001
Model 2	1.000 (Reference)	1.433 (1.088–1.887)
P-Value		0.01
Model 3	1.000 (Reference)	1.336 (1.010–1.766)
P-Value		0.042

Model 1 is adjusted for age, sex, and race. Model 2 is adjusted for variables in Model 1 + education status, smoking, drinking, poverty-income ratio, and body mass index. Model 3 is adjusted for variables in Model 2 + glycated hemoglobin, cardiovascular disease, hyperlipidemia, hypertension, chronic pulmonary disease, liver disease, arthritis, cancer, and moderate to severe nephropathy. Abbreviations: CI: confidence interval; GPS: Glasgow Prognostic Score; HR: hazard ratio.

Supplementary Table 3. Cox regression analysis for the post-hoc analysis outcomes.

Outcomes	PLR, HR (95% Cl)		NLR, HR (95% Cl)	
	0	1	0	1
Cardiovascular mortality				
Model 3	1.000 (Reference)	0.969 (0.823–1.140)	1.000 (Reference)	1.455 (1.229–1.722)
P-Value		0.704		<0.001
All-cause mortality				
Model 3	1.000 (Reference)	0.913 (0.839–0.995)	1.000 (Reference)	1.516 (1.388–1.657)
P-Value		0.037		<0.001
Cerebrovascular mortality				
Model 3	1.000 (Reference)	0.718 (0.514–1.004)	1.000 (Reference)	0.993 (0.707–1.395)
P-Value		0.053		0.968
Cancer mortality				
Model 3	1.000 (Reference)	1.003 (0.813–1.239)	1.000 (Reference)	1.314 (1.058–1.632)
P-Value		0.975		0.014

Model 3 is adjusted for age, sex, race, education status, smoking, drinking, poverty-income ratio, body mass index, glycated hemoglobin, cardiovascular disease, hyperlipidemia, hypertension, chronic pulmonary disease, liver disease, arthritis, cancer, and moderate to severe nephropathy. Abbreviations: CI: confidence interval; NLR: neutrophil-to-lymphocyte ratio; PLR: platelet-to-lymphocyte ratio; HR: hazard ratio.
